# CDC6 is a prognostic biomarker and correlated with immune infiltrates in glioma

**DOI:** 10.1186/s12943-022-01623-8

**Published:** 2022-07-25

**Authors:** Feng Wang, Fen Zhao, Li Zhang, Lai Xiong, Qing Mao, Yanhui Liu, Xiaoguang Qiu, Xiang Wang, Lin Shui, Xi Chen, Kexing Ren, Pixian Shui, Qiongwen Zhang, Yifei Deng, Weimin Li, Xiaoqi Xie, Dengbin Wu, Tao Li, Jinyi Lang, Lei Liu, Huaying Chen, Jianguo Xu, Sen Bai, Zhiping Li, Qiang Yue, Ni Chen, Bingwen Zhou, Cheng Yi, Yuquan Wei, Yuchuan Fu, Yong Luo, Qiheng Gou, Lunxu Liu, Yuanzhao Liu, Jingbo Kang, Junjie Wang, Dongcun Jing, Fuquan Zhang, Xiaoyan Yang, Xianfeng Li, Tao Jiang, Zongcun Zhang, Yizhi Zhou, Junlin Yi

**Affiliations:** 1 Department of Internal Medicine, West China Hospital Cancer Center Head And Neck, Sichuan University, Chengdu, China; 2 Department of Cancer Center Head and Neck, West China Hospital, Sichuan University, Chengdu, China; 3 Department of Oncology, Chengdu First People’s Hospital, Chengdu, 610041 Sichuan Province China; 4 Department of Radiotherapy, Beijing Tian Tan Hospital, Capital Medical University, Beijing, 100050 China; 5 Department of Pharmacy, Affiliated Hospital of Southwest Medical University, Luzhou, China; 6 Department of Radiotherapy, Chengdu Seventh Hospital, Chengdu, China; 7 Center for Precision Medicine, West China Hospital, Sichuan University, Chengdu, China; 8 Department of Critical Care Medicine, West China Hospital, Sichuan University, Chengdu, China; 9 Cancer Hospital, An Steel Group General Hospital, Anshan, Liao Ning People’s Republic of China; 10 Department of Radiotherapy, Sichuan Cancer Hospital & Institute, Sichuan Cancer Center, School of Medicine, University of Electronic Science and Technology of China, Chengdu, 610041 Sichuan China; 11 Department of Radiotherapy, Beijing Hospital, Beijing, People’s Republic of China; 12 Department of Radiotherapy, The sixth Medical Center of PLA General Hospital, Beijing, People’s Republic of China; 13 Department of Radiation Oncology, Peking University Third Hospital, No. 49, Beijing, China; 14 Kawagawa Hospital Tsukiji, Fukutai, Japan; 15 Department of Radiation Oncology, Beijing Union Medical College Hospital, Beijing, China; 16 Department of Radiotherapy, First Hospital of Shan Xi Medical, University, Taiyuan West, Taiyuan, China; 17 Qing Dao Central Hospital, 127 Si Liu South Road, Shi Bei District, Qing Dao, Shan Dong Province China; 18 Shanghai High-Tech United Bio-Technological R&D Co., Ltd, Shanghai, China; 19 Department of Radiation Oncology, National Cancer Center National Clinical Research Center for Cancer/Cancer Hospital, Chinese Academy of Medical Sciences and Peking Union Medical College, Beijing, China

**Keywords:** CDC6, Glioma, Prognosis, Immune infiltrates, Biomarker

## Abstract

**Background:**

Cell division cycle 6 (CDC6) has been proven to be associated with the initiation and progression of human multiple tumors. However, it’s role in glioma, which is ranked as one of the common primary malignant tumor in the central nervous system and is associated with high morbidity and mortality, is unclear.

**Methods:**

In this study, we explored CDC6 gene expression level in pan-cancer. Furthermore, we focused on the relationships between CDC6 expression, its prognostic value, potential biological functions, and immune infiltrates in glioma patients. We also performed vitro experiments to assess the effect of CDC6 expression on proliferative, apoptotic, migrant and invasive abilities of glioma cells.

**Results:**

As a result, CDC6 expression was upregulated in multiple types of cancer, including glioma. Moreover, high expression of CDC6 was significantly associated with age, IDH status, 1p/19q codeletion status, WHO grade and histological type in glioma (all *p* < 0.05). Meanwhile, high CDC6 expression was associated with poor overall survival (OS) in glioma patients, especially in different clinical subgroups. Furthermore, a univariate Cox analysis showed that high CDC6 expression was correlated with poor OS in glioma patients. Functional enrichment analysis indicated that CDC6 was mainly involved in pathways related to DNA transcription and cytokine activity, and Gene Set Enrichment Analysis (GSEA) revealed that MAPK pathway, P53 pathway and NF-κB pathway in cancer were differentially enriched in glioma patients with high CDC6 expression. Single-sample gene set enrichment analysis (ssGSEA) showed CDC6 expression in glioma was positively correlated with Th2 cells, Macrophages and Eosinophils, and negative correlations with plasmacytoid dendritic cells, CD8 T cells and NK CD56bright cells, suggesting its role in regulating tumor immunity. Finally, CCK8 assay, flow cytometry and transwell assays showed that silencing CDC6 could significantly inhibit proliferation, migration, invasion, and promoted apoptosis of U87 cells and U251 cells (*p* < 0.05).

**Conclusion:**

In conclusion, high CDC6 expression may serve as a promising biomarker for prognosis and correlated with immune infiltrates, presenting to be a potential immune therapy target in glioma.

**Supplementary Information:**

The online version contains supplementary material available at 10.1186/s12943-022-01623-8.

## Background

Glioma, accounting for almost 80% of malignant brain tumors, is the most common primary malignant tumor in central nervous system (CNS) with high degree of mortality and malignancy [1]. The current standard regimens for glioma are mainly focused on maximizing safe surgical resection of tumor and assisting in adjuvant radiotherapy and chemotherapy, while high frequencies of recurrence and metastasis remain a huge challenge [2]. In recent years, although the application of immunotherapy, targeted therapy, photodynamic therapy and electric field therapy have improved the prognosis of glioma patients, the effects remain far from satisfactory [3]. For these reasons, the identification of key molecules involved in glioma is urgent and highly demanded for improving the clinical outcome.

Cell division cycle 6 (CDC6), mapped to chromosome 17q21.3, is an essential licensing factor for DNA replication during G1 phase and S phase in eukaryotic cells [4]. CDC6 also plays crucial roles in the development and maintenance of the S-M phase checkpoint mechanisms in the cell cycle [5]. Furthermore, many previous studies have shown that abnormal expression of CDC6 is involved in oncogenic activities in a variety of malignancies, and may be a potential diagnostic and prognostic marker for related tumors. For example, Kim et al. determined that elevated expression of CDC6 was highly correlated with poor prognosis of prostate cancer (PCa) [6]. Mahadevappa et al. reported that expression of CDC6 was significantly higher in breast cancer, especially in estrogen receptor (ER) negative breast cancer, suggesting that it may be a potential prognostic marker and therapeutic target in breast cancer patients [7]. While its role in glioma is unclear. Therefore, we aimed to demonstrate the relationship between CDC6 expression and glioma.

To clarify the role of CDC6 in glioma, we explored the relationship between CDC6 expression and glioma. Our results revealed that CDC6 was upregulated in glioma tissues and high CDC6 expression was correlated with poor prognosis and immune infiltrates in glioma patients. These findings suggest that CDC6 may be a potentially promising target by regulating its interaction with infiltrating immune cells in glioma patients.

## Results

### Clinical characteristics of the glioma patients

The microarray data of GSE104291 was downloaded from The Gene Expression Omnibus (GEO) database involving 4 glioma samples and 2 normal samples, while detailed clinical information were not available.

The RNA sequencing data across 33 tumor types and normal tissues of 118,103 samples were downloaded from The Cancer Genome Atlas (TCGA) and Genotype-Tissue Expression (GTEx) database, including glioma (contained 689 tumor samples and 1157 normal samples). A total of 298 female and 398 male patients were involved in the present study, of which 20.5% (*n* = 143) were over age 60. As for race, most of the cases 637 (93.3%) were white. The WHO grade included 224 (35.3%) G2, 243(38.3%) G3 and 168 (26.5%) G4. IDH (isocitrate dehydrogenase) status involved 246 (35.9%) with IDH-wide-type (IDH-WT) and 440 (64.1%) with IDH-mutant (IDH-MT). 1p/19q codeletion status included 171 (24.8%) codel and 518 (75.2%) non-codel. In terms of primary therapy outcome, 112 (24.2%) were progressive disease (PD), 147 (31.8%) patients were stable disease (SD), 64 (13.9%) patients were partial response (PR), and 139 (30.1%) patients were complete response (CR). Besides, as for histological type, 195(28%) of patients were astrocytoma, 134 (19.3%) of patients were oligoastrocytoma, 199 (28.6%) of patients were oligodendroglioma and 168 (24.1%) were glioblastoma.

From The Chinese Glioma Genome Atlas (CGGA), 325 tumors with both gene expression data and clinical futures were analyzed. The clinical characteristics of the glioma patients including age, gender, histology, grade, IDH status, 1p/19q codeletion status, chemo-status, radio-status, and MGMTp_methylation status and etc. Among the 325 participants, 203 (62%) were male and 122 (38%) were female, and the median age of all participants was 40.5 years.

### Abnormally high expression of CDC6 in glioma

**Fig. 1:**
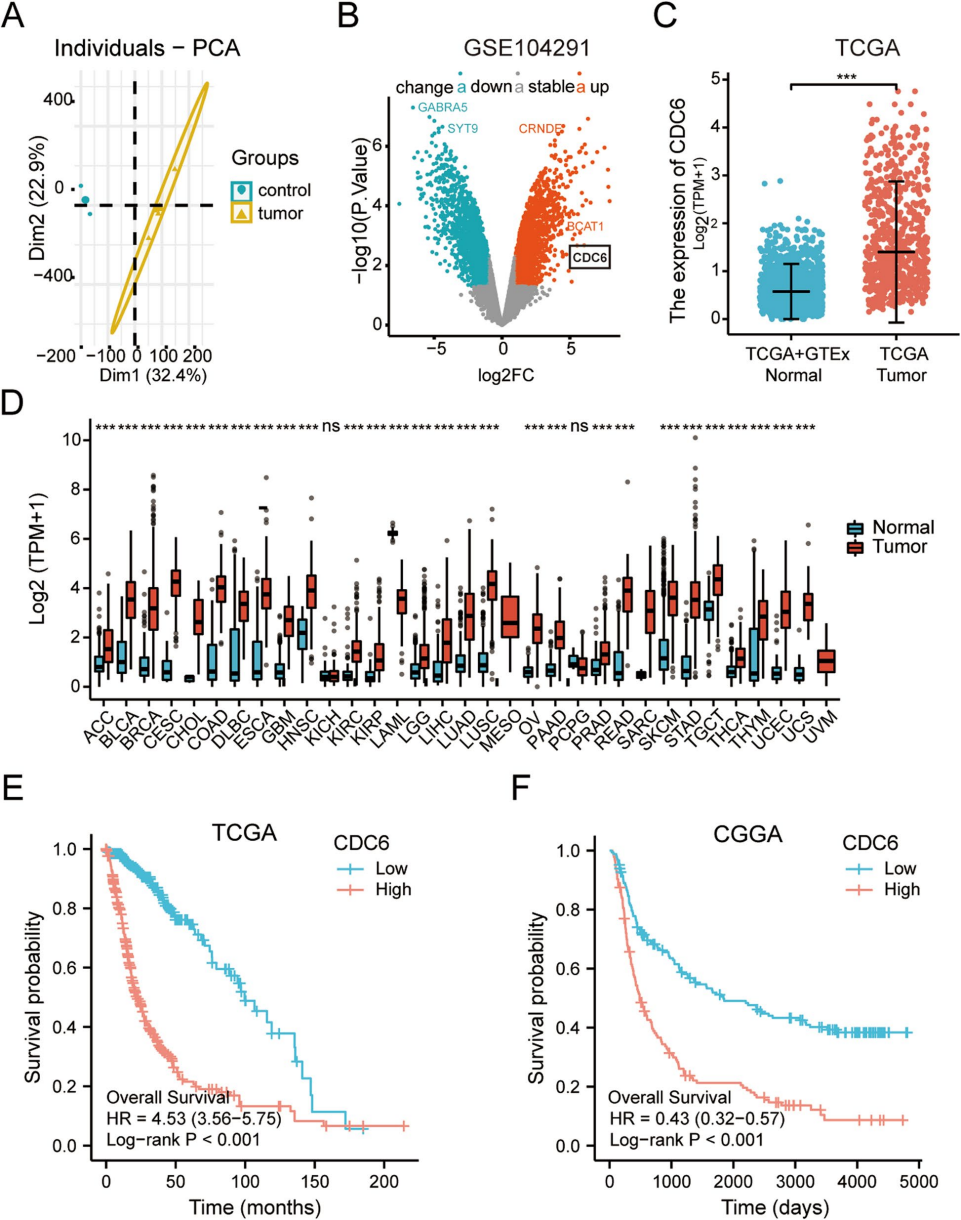
The expression of CDC6 in different human cancers and its relationship with glioma prognosis. **A** PCA plot of the data showing no batch effect in the GSE104291 dataset. Red nodes represent the tumor cluster while blue nodes represent the normal cluster. **B** The volcano plots of DEGs in GSE104291. Red nodes represent upregulated genes, blue nodes represent downregulated genes, and gray indicates genes with no differential expression based on the criteria of *p* value < 0.05 and |log2 FC| > 1, respectively. **C** Wilcoxon rank sum test was used to analyze the differential expression of CDC6 in glioma tissues and normal tissues with the data of the GTEx database as controls. **D** CDC6 expression in TCGA tumors and normal tissues with the data of the GTEx database as controls. **E** Kaplan–Meier analysis of association between CDC6 expression and glioma prognosis in TCGA. **F** Kaplan–Meier analysis of association between CDC6 expression and glioma prognosis in CGGA. PCA, principal component analysis; DEGs, differentially expressed genes; log2 FC, log2 foldchange; TCGA, The Cancer Genome Atlas; GTEx, Genotype-Tissue Expression; CGGA, Chinese Glioma Genome Atlas. ns, *p* ≥ 0.05, *p < 0.05, **p < 0.01, ***p < 0.001

As shown in Fig. 1A, principal component analysis (PCA) plot showed that no batch effect was found in the GSE10429 dataset. After the R language limma package processed, a total of 3742 differentially expressed genes (DEGs) were identified between glioma tissues and normal tissues, including 2179 downregulated genes and 1563 upregulated genes. The volcano plots presented the expression of DEGs (Fig. 1B), and among them, the expression level of CDC6 was significantly upregulated in the GSE10429 dataset.

We analyzed the expression of CDC6 in 689 glioma samples of TCGA database and 1157 normal samples of TCGA database combined GTEx database, and found that CDC6 was significantly high expressed in glioma samples (Fig. 1C; *p* < 0.001). We further expanded the number of samples to evaluate the mRNA expression level of CDC6 across pan-cancer in TCGA tumors with the data of the GTEx database as controls. As shown in Fig. 1D, compared with normal tissues, CDC6 was significantly upregulated in 28 of 33 cancer types, including bladder urothelial carcinoma (BLCA), breast invasive carcinoma (BRCA), glioblastoma multiforme (GBM), head and neck squamous cell carcinoma (HNSC), kidney renal clear cell carcinoma (KIRC), renal papillary cell carcinoma (KIRP), brain lower grade glioma (LGG), lung adenocarcinoma (LUAD), lung squamous cell carcinoma (LUSC), ovarian serous cystadenocarcinoma (OV), prostate adenocarcinoma (PRAD), stomach adenocarcinoma (STAD), oral squamous cell carcinoma (OSCC) and so on (all *p* < 0.05), while it was downregulated in acute myeloid leukemia (LAML) (*p* < 0.05), no difference was found in kidney chromophobe (KICH), pheochromocytoma and paraganglioma (PCPG), mesothelioma (MESO) and uveal melanoma (UVM). The results indicated that the mRNA expression of CDC6 was also highly expressed across multiple types of cancer.

### Relationship between expression of CDC6 and survival in glioma

**Fig. 2:**
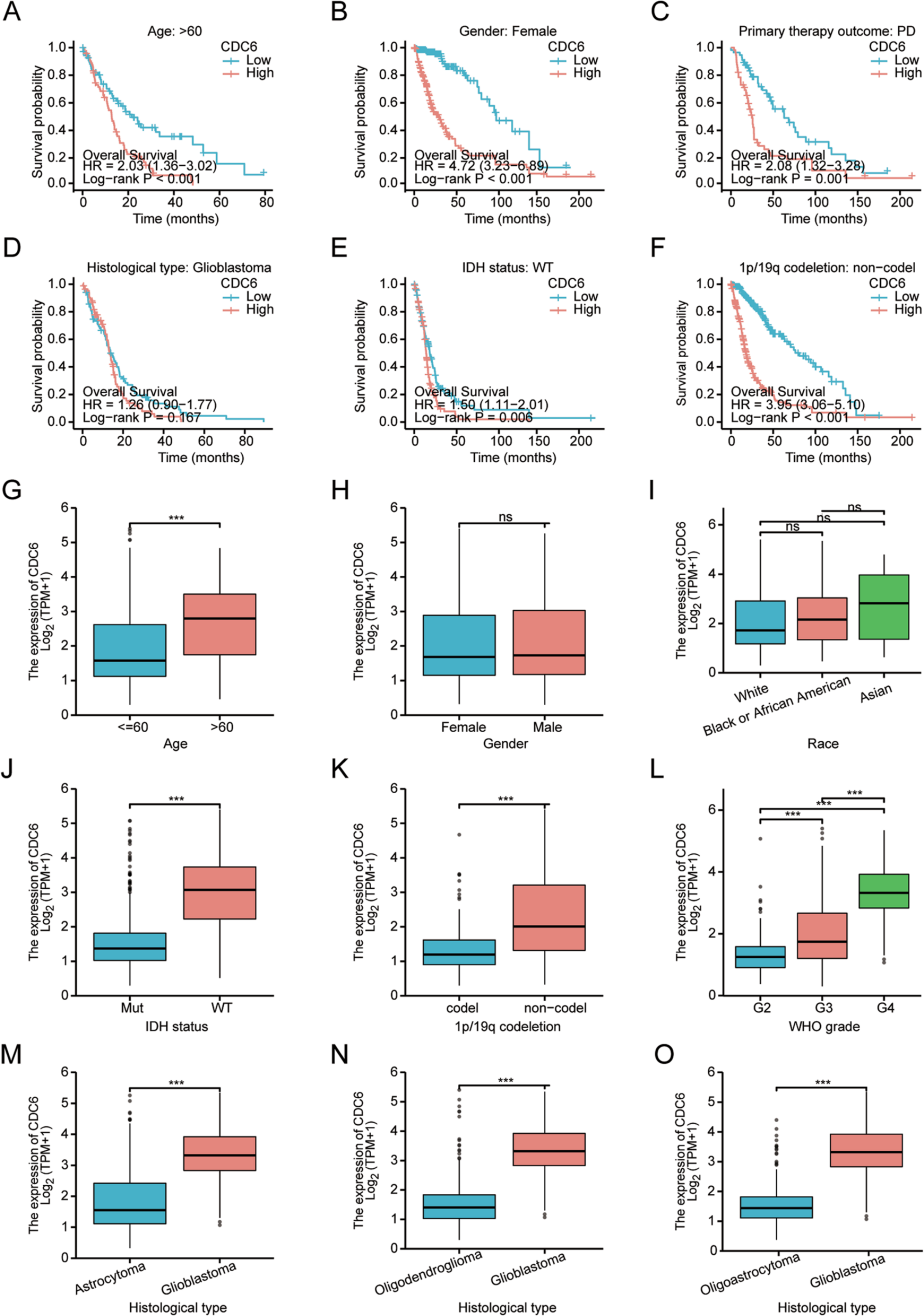
Transcriptional expression level and prognosis value of CDC6 in glioma from TCGA database. **A-I** The mRNA expression levels of CDC6 were analyzed in glioma patients according to (**A**) Age, (**B**) Gender, (**C**) Race, (**D**) IDH status, (**E**) 1p/19q codeletion, (**F**) WHO grade, (**G-I**) histological type, respectively. **J-O** Overall survival analysis towards the CDC6 expression was performed in subgroups of glioma patients: (**J**) Age: > 60, (**K**) Gender: Female, (**L**) primary therapy outcome: PD, (**M**) histological type: Glioblastoma, (**N**) IDH status: WT, (**O**) 1p/19q codeletion: non-codel. TCGA, The Cancer Genome Atlas; IDH, isocitrate dehydrogenase; WHO, World Health Organization; PD, progressive disease; WT, wild-type. ns, *p* ≥ 0.05, ***p < 0.001

Kaplan-Meier analysis revealed that glioma patients from the TCGA database with high CDC6 expression was correlated with poor OS (Fig. 1E; *p* < 0.001). Then, similar analysis in glioma patients from the CGGA dataset demonstrated that OS was significantly decreased in patients with high CDC6 mRNA expression compared with those with low CDC6 mRNA expression (Fig. 1F; *p* < 0.001). Furthermore, we investigated the correlations between CDC6 expression and prognosis (OS) in different clinical subgroups of glioma from TCGA database. The results showed that the higher expression of CDC6 had a worse OS in following clinical subgroups, including subgroup of age > 60 years (Fig. 2A; *p* < 0.001), subgroup of female (Fig. 2B; *p* < 0.001), subgroup of primary therapy outcome (PD) (Fig. 2C; *p* < 0.001), subgroup of IDH status (WT) (Fig. 2E; *p* < 0.01) and subgroup of 1p/19q codeletion status (non-codel) (Fig. 2F; *p* < 0.001). However, no difference in OS was observed upon histological type (Glioblastoma) (Fig. 2D; *p* = 0.167).

### Association between CDC6 expression and clinicopathological features of glioma patients

To evaluate the association between CDC6 expression and clinicopathological features of glioma patients, the clinical characteristics of 689 glioma patients including RNA sequencing data from TCGA database were analyzed. Based on the mean value of CDC6 expression, the patients were divided into high- and low- CDC6 expression groups, we performed Wilcoxon rank-sum test, Kruskal-Wallis test and logistic regression analysis. As shown in Fig. 2G-O, Our results showed that higher expression levels of CDC6 were observed in patients with age > 60 years (Fig. 2G; *p* < 0.001), IDH status (WT) (Fig. 2J; *p* < 0.001), 1p/19q codeletion status (non-codel) (Fig. 2K; *p* < 0.001), WHO grade (G3/G4) (Fig. 2L; *p* < 0.001), and worse histological type (Fig. 2M-O; *p* < 0.001). However, no statistically significant correlation was found between the expression levels of CDC6 and other clinical pathological characteristics, such as gender (Fig. 2H; *p* ≥ 0.05) and race (Fig. 2I; *p* ≥ 0.05). These data indicate that high expression of CDC6 is significantly associated with age, IDH status, 1p/19q codeletion status, WHO grade and histological type, respectively.

Univariate analysis using logistic regression demonstrated that CDC6 expression was correlated with poor prognostic features in glioma patients. High CDC6 expression was significantly correlated with age (> 60 vs. <= 60: OR = 4.212, 95%CI = 2.795–6.490, *p* < 0.001), WHO grade (G4&G3 vs. G2: OR = 9.413, 95%CI = 6.408–14.077, *p* < 0.001), IDH status (WT vs. MT: OR = 15.032, 95%CI = 10.032–23.106, *p* < 0.001), 1p/19q codeletion status (non-codel vs. codel: OR = 5.413, 95%CI = 3.638–8.230, *p* < 0.001), primary therapy outcome (PD vs. CR&PR&SD: OR = 2.656, 95%CI = 1.720–4.118, *p* < 0.001) and histological type (glioblastoma vs. astrocytoma&oligoastrocytoma&oligodendroglioma: OR = 31.688, 95%CI = 16.724–68.190, *p* < 0.001). However, there was no significant difference in overall survival (OS) upon gender and race (all *p* > 0.05). Taken together, these results indicate that the glioma patients with high CDC6 expression are associated with worse clinicopathological characteristics and may serve as a biomarker of poor prognosis.

### Cox univariate and multivariate analysis of prognostic factors in glioma

Univariate and multivariate Cox proportional hazard regression analyses were carried out with glioma patients from TCGA database. The univariate analysis indicated that high CDC6 expression was associated with the worse OS (HR = 4.608; *p* < 0.001). Other clinical parameters, such as gender (HR = 1.262; *p =* 0.062), age (HR = 4.668; *p* < 0.001), WHO grade (HR = 18.615; *p* < 0.001), IDH status (HR = 4.608; *p* < 0.001), 1p/19q codeletion status (HR = 4.428; *p* < 0.001), primary therapy outcome (HR = 3.542; *p* < 0.001) and histological type (HR = 9.114; *p* < 0.001) were also correlated with the worse OS time. Following multivariate Cox analysis, the results showed that gender (HR = 1.945; *p* = 0.004), age (HR = 4.689; *p* < 0.001), WHO grade (HR = 4.879; *p* = 0.006), IDH status (HR = 0.544; *p* = 0.026) and primary therapy outcome (HR = 3.643; *p* < 0.001) were independent prognostic factors in OS of glioma patients. However, we could not exhibit statistical significance of CDC6 expression in OS by multivariate Cox analysis (HR = 1.470; *p* = 0.078).

### Functional enrichment analysis of DEGs

To elucidate the biological functions of CDC6, we analyzed the DEGs between high- and low- CDC6 expression groups based on the median CDC6 expression level. A total of 1357 DEGs were acquired with the threshold values of adjusted *p* value (*p*.adj) < 0.05 and |log2 FC| > 2, including 1321 upregulated genes and 36 downregulated genes, that were presented in volcano plots. Then, we conducted Gene Ontology (GO) and Kyoto Encyclopedia of Genes and Genomes (KEGG) enrichment analyses of DEGs, revealing that the primary biological process (BP) contained pattern specification process, embryonic organ development, regionalization, and anterior/posterior pattern specification. The cellular component (CC) was mainly enriched in collagen-containing extracellular matrix, kinetochore, condensed chromosome, centromeric region, and DNA packaging complex. The molecular function (MF) was primarily involved in receptor ligand activity, DNA-binding transcription activator activity, RNA polymerase II-specific, cytokine activity, and extracellular matrix structural constituent. Further functional enrichment analyses showed significantly enriched KEGG pathway in cytokine-cytokine receptor interaction, transcriptional misregulation in cancer, cell cycle, systemic lupus erythematosus and ECM-receptor interaction.

### GSEA identifies CDC6-related signaling pathway

To identify CDC6-related signaling pathways in glioma, GSEA between high- and low- CDC6 expression datasets was conducted to reveal significant differences (*p*. adj < 0.05, false discovery rate (FDR) < 0.25) in enrichment of the Molecular Signatures Database (MSigDB) Collection (c5.bp.v7.2.symbols.gmt (Gene ontology) and c2.cp.v7.2.symbols.gmt (Curated)). In all, 3 GO items including single organism behavior, gated channel activity, cognition, transporter complex and ligand gated channel activity were showed significantly differential enrichment in CDC6 high expression phenotype. The results showed that the biological processes strongly associated with CDC6 were cell proliferation and immune-related pathways. 6 KEGG items that exhibited significantly differential enrichment in the CDC6-high expression phenotype were identified, including MAPK activation, DNA repair, P53 signaling pathway, focal adhesion, core matrisome and NF-κB activation. The results revealed that these pathways positively associated with CDC6 were responsible for regulation of cell proliferation/apoptosis and tumor invasion. Taking together, these findings suggest that these biological processes and signaling pathways of CDC6-high expression, which are critically important in development and metastasis of tumor, may be a potential target for the treatment of glioma.

### The correlation between CDC6 expression and immune cell infiltration

We further explored the association between the expression level of CDC6 and immune cell infiltration level quantified by ssGSEA in glioma using Spearman correlation. The results showed that CDC6 expression was positively correlated with infiltration levels of Th2 cells, Macrophages, Eosinophils, etc., and negatively correlated with that of plasmacytoid dendritic cells (pDCs), natural killer (NK) CD56 bright cells, etc.

### CDC6 promotes proliferation and inhibits apoptosis in glioma cells

To explore the effect of CDC6 on glioma progression, U87 and U251 cells were transfected with sh-CDC6–1004, and the transfection efficiency was confirmed by western blot analysis. The results showed that the expression levels of protein in the sh-CDC6–1004 group was significantly lower than that in the sh-NC group. CCK-8 was used to assess role of CDC6 on glioma cell proliferation, and the results showed that the proliferative viability of U87 and U251 cells in the sh-CDC6–1004 group was smarkedly lower than that in the sh-NC group, particularly at 72 h. Flow cytometry was further performed to analyze apoptosis of the transfected cells, the results indicated that, compared with the sh-NC group, the sh-CDC6–1004 group showed greater apoptosis of U87 and U251 cells. Our results suggest that CDC6 promotes proliferation and inhibits apoptosis of glioma cells.

### CDC6 facilitates migration and invasion of glioma cells

To further clarify whether CDC6 affects the ability of migration and invasion of glioma cells, we performed the transwell assays. The results showed that the number of migrated and invaded U87 and U251 cells in the sh-CDC6–1004 group was smarkedly lower than that in the sh-NC group. These findings reversely suggest that CDC6 promotes migration and invasion of glioma cells.

## Discussion

CDC6, DNA replication factor, which associates with DNA replication origins and is required for replication initiation; hence, it is closely associated with tumorigenesis and development. In recent years, many studies about association between CDC6 expression and prognosis of multiple tumors have emerged recently. For example, Zhang et al. found that CDC6 was aberrantly expressed in lung cancer tissues, and overexpression of CDC6 was associated with poor OS of lung cancer patients [8]. Mahadevappa et al. reported that high level of CDC6 expression was significantly associated with a poorer survival time in ER positive breast cancer [7]. Kim et al. determined that CDC6 mRNA expression was significantly higher in PCa tissues than that in controls and elevated expression of CDC6 was highly correlated with poor prognosis of patients with PCa [6]. Consistently, here we found that glioma patients with high CDC6 expression had a worse prognosis than those with low CDC6 expression, especially in certain clinical subgroups, such as age > 60 years, female, primary therapy outcome (PD), IDH status (WT) and 1p/19q codeletion status (non-codel). In subgroup of Glioblastoma, our results showed that CDC6 was highly expressed in tumor tissues, while survival analysis showed no significant correlation between the expression level of CDC6 and prognosis of patients. Considering that there were only 168 glioblastomas in this subgroup, the sample size was small and may not have sufficient statistical efficacy, and the conclusions may be biased. Moreover, CDC6 expression level as a risk factor in OS of glioma through univariate Cox regression analyses, and by multivariate Cox analysis, we could not exhibit statistical significance of CDC6 expression in OS. Considering the current data derived from the TCGA public database, retrospective studies still have their own bias due to potential confounding factors. Therefore, a prospective study should be performed to verify the prognostic predictive role of CDC6 in glioma in the future. The results are further supported by the prior research involving prognostic significance of abnormally high expression of CDC6 in GBM [9], which identified high CDC6 expression as prognostic variables for the OS. These results together thus suggest that CDC6 may have value as a glioma prognostic biomarker.

Many studies about biological function and signaling pathways of glioma have emerged recently. Wang et al. and Li et al. reported that enhanced activity of NF-κB signaling pathway promoted growth of GBM in vivo and induced ferroptosis of glioma cell lines [10]. Krex et al. reported that the p53 pathway was highly deregulated in GBM, the mutational status of TP53 was related with GBM growth [11]. Through GSEA, our study revealed that NF-κB signaling pathway, MAPK pathway and P53 pathway were differentially enriched in glioma patients with high CDC6 expression phenotype, indicating that CDC6 may promote glioma cell growth and migration via these pathways. Those all suggest that CDC6 may serve as a potential therapeutic target in glioma.

Tumor-infiltrating immune cells (TIICs) are indispensable component of the tumor microenvironment (TME), which play an important role in the growth and progression of tumors. In recent years, a large number of studies about the possible role of CDC6 in human TIICs have emerged. Cong et al. reported that CDC6 was related to B cell, T cell infiltration, macrophage infiltration and dendritic cell (DC) infiltration, and may be a prognostic factor for Clear Cell Renal Cell Carcinoma patients [12]. However, the correlation between CDC6 expression and immune cell infiltration in glioma has not been investigated. In present study, the results revealed that CDC6 expression was positively correlated with infiltration abundances of Th2 cells, Macrophages and Eosinophils, and negatively correlated with that of pDCs, CD8 T cells and NK CD56bright cells. These correlations could be indicative of a potential mechanism by which CDC6 inhibits the function of pDCs, CD8 T cells and NK CD56bright cells, subsequently promotes the function of Th2 cells, Macrophages, and Eosinophils, which are responsible for maintaining the immunosuppressive local microenvironment of tumor, and thus contribute to the development and progression of tumor. These findings suggest that CDC6 may be a potentially promising target by regulating its interaction with infiltrating immune cells in glioma patients. However, the detailed underlying mechanisms still need to be further explored.

Abnormal and unrestricted cell growth is a hallmark of cancer and is caused by the misregulation of various crucial protein expression, which leads to the occurrence, progression and recurrence of glioma patients [13]. CDC6, a chromosome replication licensor in the cell cycle, plays a major role in cell proliferation, migration, invasiveness and tumor metastasis in several cancers. Deng et al. demonstrated that CDC6 protein level was highly expressed in ovarian cancer tissues, silencing CDC6 decreased cell proliferation and colony formation in HO8910 cells [14]. Zhao et al. found that CDC6 was highly expressed in gastric cancer cells, down-regulation of CDC6 inhibited cell proliferation, invasion, and promoted apoptosis of BGC823 and SGC7901 cell lines [15]. The present study demonstrated that the CDC6 protein level in U87 and U251 cells was higher than that in normal cell, and silencing CDC6 inhibited proliferation, migration and invasion, and promoted apoptosis of glioma cells. The result suggests that CDC6 may act as a novel oncogenic in glioma and may be a potential therapeutic target.

Although the present study suggested some correlations between CDC6 expression and glioma, there are still some limitations in this study. Firstly, number of healthy samples used as controls differed significantly from that of tumor samples, hence additional studies were required to maintain a balance of sample size. Secondly, the current study was performed primarily based on bioinformatic analyses and in vitro experiments, further more clinical samples of glioma patients are required to verify the abnormally expression of CDC6. Meanwhile, it is necessary to further elucidate the biological functions of CDC6 in glioma and the underling molecular mechanisms in subsequent experiments. Last but not most, retrospective studies still have their own bias caused by potential confounding factors because of data from public databases, especially non-uniform intervening measures and lacking of some detailed information; herefore, a prospective study should be performed in the future to avoid analysis bias.

## Conclusion

Our results showed that CDC6 expression was highly expressed in glioma tissues, and high CDC6 expression was significantly correlated with poor survival and immune infiltration of glioma, which might promote tumor tumorigenesis through abnormal inflammation and immune response of glioma patients. In addition, CDC6 was mainly enriched signaling pathways such as NF-κB signaling pathway, MAPK pathway and P53 pathway, therefore CDC6 may be involved in the progression of glioma. We also found that CDC6 promotes the proliferative, migrant and invasive capabilities of glioma cells, and inhibits apoptosis of glioma cells. Collectively, our results suggest that CDC6 may serve as a promising biomarker for prognosis in glioma and correlated with immune infiltrates, presenting to be a potential immune therapy target in glioma.

## Supplementary Information

**Additional file 1.**

## Data Availability

The results within this publication are in part based upon data generated by the GEO database (http://www.ncbi.nlm.nih.gov/geo), TCGA and GTEx database by UCSC XENA (https://xenabrowser.net/datapages/), and CGGA database (http://www.cgga.org.cn/). In addition, All data used and/or analyzed during the current study are available from the corresponding author on reasonable request.

## Abbreviations

CDC6: Cell division cycle 6
CGGA: The Chinese Glioma Genome Atlas
CNS: central nervous system
CR: complete response
DEGs: differentially expressed genes
FDR: false discovery rate
GBM: glioblastoma
GO: Gene Ontology
GEO: The Gene Expression Omnibus
GSEA: Gene Set Enrichment Analysis
GTEx: Genotype-Tissue Expression
HR: hazard ratio
IDH: isocitrate dehydrogenase
KEGG: Kyoto Encyclopedia of Genes and Genomes
MSigDB: Molecular Signatures Database
NES: Normalized enrichment scores
NK: natural killer
OS: overall survival
p.adj: adjusted *p* value
PCA: principal component analysis
PD: progressive disease
pDCs: plasmacytoid dendritic cells
PR: partial response
SD: stable disease
ssGSEA: Single-sample gene set enrichment analysis
TCGA: The Cancer Genome Atlas
TIICs: tumor-infiltrating immune cells
TME: tumor microenvironments
WB: Western blot
